# Plasma in Cancer Treatment

**DOI:** 10.3390/cancers12092617

**Published:** 2020-09-14

**Authors:** Angela Privat-Maldonado, Annemie Bogaerts

**Affiliations:** PLASMANT, Chemistry Department, University of Antwerp, 2610 Antwerp, Belgium

Cancer is the second leading cause of death worldwide, and while science has advanced significantly to improve the treatment outcome and quality of life in cancer patients, there are still many issues with the current therapies, such as toxicity and the development of resistance to treatment. The scientific community conducting oncological research is putting significant efforts into finding new and efficient alternatives in order to reduce the harmful side effects caused by conventional cancer therapies. One of these is cold atmospheric plasma (CAP), which involves the application of an ionized gas, rich in ions, electrons, radicals and excited species, able to eliminate cancerous cells and contribute to healing cancerous lesions [1,2]. Compared to traditional systemic anticancer therapies, CAP can be administered locally and can modulate and activate multiple signaling pathways in cancer cells, which contribute to their elimination [3]. Exciting advances made in the past few years in the field of biomedical plasma have allowed scientists to explore its use in different types of cancer. To date, some of the key events involved in the response to CAP-derived reactive oxygen species (ROS), such as cell death, senescence and cell cycle arrest, among others [4,5,6], have been identified in cancer cells. However, the response evoked by CAP in different populations of cells (cancerous, stromal, immune cells) varies greatly and selectivity studies could help to unravel this issue. In addition, it is important to consider the three-dimensional nature of solid tumors, where the tumor microenvironment plays an important role in the response to therapy [7]. 

The scope of CAP for cancer therapy is rapidly expanding to address difficult targets which were previously untreatable, including those with metastatic potential and resistance to drugs. To progress towards a widespread clinical application of CAP, an integrated study of the multi-dimensional effect of CAP in cancer treatment is essential. 

This Special Issue on “Plasma in Cancer Treatment” brings together 16 original research papers [8,9,10,11,12,13,14,15,16,17,18,19,20,21,22,23] and two insightful reviews [24,25]. The papers published in the Special Issue provide valuable information regarding the efficacy of CAP against osteosarcoma, glioblastoma, cholangiocarcinoma, melanoma, pancreatic, ovarian, breast, cervical and colorectal cancer. The article collection includes studies on the fundamental mechanisms of action during oxidative stress and chemotherapy [12], molecular mechanisms of action [24], cell cycle regulation [25], activation of cell signaling pathways [14], effect on stromal and immune cells [8,17], metastatic potential [23], the tumor microenvironment [17,25] and selectivity of CAP towards cancer cells [22]. CAP has been used in combination with chemotherapeutics and radiation therapy to boost their cytotoxic activity [9,10] and to restore sensitivity to chemotherapeutics [11]. In combination with low pulse electric fields, CAP improves the permeabilization of cells [19], which could be beneficial for drug delivery. The addition of gold quantum dots to CAP treatment can further boost the efficacy of the treatment [13]. In addition, the use of non-thermally operated electrosurgical argon plasma devices for cancer therapy has been explored [15,16], which presents an opportunity to use existing devices for cancer treatment. Three reports have used plasma-treated Ringer’s saline and phosphate buffered-saline (PBS) solutions with anticancer properties, supporting the potential of this alternative treatment modality [18,20,21]. Two review papers complete this Special Issue. The first summarizes the current state of knowledge on the molecular mechanisms of action of CAP [24] and the second explores the role of the tumor microenvironment in the response to CAP treatment and presents useful three-dimensional in vitro culture models for plasma research [25]. 

In summary, this Special Issue presents the effect of CAP on a wide range of cancer types, highlighting the versatility of CAP and its future application in the field. The studies presented here offer an opportunity to consider the application of CAP in the clinic to improve survival rates and quality of life of cancer patients in the near future.
